# Curcumin alleviates diabetic nephropathy via inhibiting podocyte mesenchymal transdifferentiation and inducing autophagy in rats and MPC5 cells

**DOI:** 10.1080/13880209.2019.1688843

**Published:** 2019-11-19

**Authors:** Qiudi Tu, Yiwen Li, Juan Jin, Xinxin Jiang, Yan Ren, Qiang He

**Affiliations:** a Department of Nephrology, Zhejiang Provincial People’s Hospital, Hangzhou, China;; b People’s Hospital of Hangzhou Medical College, Hangzhou, China;; c Key Laboratory of Kidney Disease of Traditional Chinese Medicine in Zhejiang Province, Hangzhou, China

**Keywords:** Glomerular podocytes, epithelial-to-mesenchymal transition, P13K/Akt, mTOR

## Abstract

**Context:** Curcumin could ameliorate diabetic nephropathy (DN), but the mechanism remains unclear.

**Objective:** The efficacy of curcumin on epithelial-to-mesenchymal transition (EMT) of podocyte and autophagy *in vivo* and *in vitro* was explored.

**Materials and methods:** Thirty male Sprague–Dawley rats were divided into the normal, model and curcumin (300 mg/kg/d, i.g., for 8 weeks) groups. Rats received streptozotocin (50 mg/kg, i.p.) and high-fat-sugar diet to induce DN. Biochemical indicators and histomorphology of renal tissues were observed. In addition, cultured mouse podocytes (MPC5) was induced to EMT with serum from DN rats, and then exposed to curcumin (40 µM) with or without fumonisin B1, an Akt specific activator or 3BDO, the mTOR inducer. Western blot analysed the levels of EMT and autophagy associated proteins.

**Results:** Administration of curcumin obviously reduced the levels of blood glucose, serum creatinine, urea nitrogen and urine albumen (by 28.4, 37.6, 33.5 and 22.4%, respectively), and attenuated renal histomorphological changes in DN rats. Podocytes were partially fused and autophagic vacuoles were increased in curcumin-treated rats. Furthermore, curcumin upregulated the expression of E-cadherin and LC3 proteins and downregulated the vimentin, TWIST1, p62, p-mTOR, p-Akt and P13K levels in DN rats and MPC5 cells. However, fumonisin B1 or 3BDO reversed the effects of curcumin on the expression of these proteins in cells.

**Discussion and conclusions:** The protection against development of DN by curcumin treatment involved changes in inducing autophagy and alleviating podocyte EMT, through the PI3k/Akt/mTOR pathway, providing the scientific basis for further research and clinical applications of curcumin.

## Introduction

Diabetic nephropathy (DN) is a common diabetic microvascular complication and one of the main causes of end-stage renal disease (ESRD), which seriously endangers human health (Wu et al. 2018). The World Health Organization’s (WHO) latest estimate is that there were about 422 million people with diabetes worldwide in 2014, which is expected to increase to 600 million by 2040 (WHO 2016), and about 1/3 of them will develop DN (Marikanty et al. 2016). Podocytes, or glomerular visceral epithelial cells, together with the glomerular basement membrane and the glomerular endothelial cells, constitute the glomerular filtration barrier. DN is closely related to the damage of glomerular podocytes, which are damaged at the early stage of diabetes. Changes in the morphology, number and density of the foot processes, expression of related proteins, and other aspects lead to the occurrence and development of DN. Under the effect of certain stimuli, glomerular podocyte would appear the phenotypic change of epithelial-to-mesenchymal transition (EMT), and loss of podocyte-specific markers, showing the characteristics of trans-differentiation. Podocytes get automaticity after EMT, fall off the glomerular basement membrane and then be flushed into the urine and excreted (Simonson 2007). Mounting evidence shows that EMT is an important mechanism that generates myofibroblasts in DN (Wang et al. 2016; Huang et al. 2017).

Autophagy is a double-edged sword, and its excessive activation or inhibition becomes the cause of injury to podocytes. Insufficient autophagy in podocytes was observed in patients with diabetes and massive proteinuria. Impaired autophagy activity contributes to DN pathogenesis and restoration of autophagy activity may be a promising therapeutic target of DN (Kim et al. 2018; Wu F et al. 2018). Currently, the latest studies have confirmed that the PI3k/Akt/mTOR signalling pathway is an important pathway widely existing in podocytes to regulate autophagy (Wang et al. 2019).

Curcumin, the major active component of turmeric, possesses extensive known pharmacological properties, including anti-inflammatory, antioxidant, and antitumor effects. Increasing evidence suggested that curcumin may offer protection against diabetic complications (Kim et al. 2016; Parsamanesh et al. 2018). It was reported that renoprotective effect of curcumin appeared to be mediated by the inhibition of NOD-like receptor 3 (NLRP3) inflammasome activity and was a promising treatment for DN (Lu et al. 2017). In addition, curcumin suppressed advanced glycation or glycoxidation end product induced apoptosis in tubular epithelial cells via protective autophagy (Wei et al. 2017).

In this study, we demonstrated that curcumin could reduce the levels of some biochemical indexes, improve the renal pathological changes, regulate autophagy and inhibit mesenchymal trans-differentiation in experimental DN rats and MPC5 cells.

## Materials and methods

### Experimental animals

Male Sprague–Dawley rats weighing 180 ± 20 g at 5 weeks of age (*n* = 30) were obtained from the Animal Center of Zhejiang Chinese Medical University (Zhejiang, China) and housed in a 12 h light/dark cycle at 22 ∼ 24 °C with free access to water and food. The protocols for animal experimentation and the care of animals were consistent with the licenses held by the Zhejiang Chinese Medical University, which fulfil and follow international rules and guidelines.

### Type 2 DN induction and treatment

SD rats were randomly divided into three groups (*n* = 10 in each group): the normal group rats received normal diet; model group and curcumin group received high-fat-sugar diet. Streptozocin (STZ, 50 mg/kg, Sigma-Aldrich, St. Louis, MO, USA) in 0.05 mol/L citrate buffer (pH = 4.5) was intraperitoneally injected to rats after treatment with high-fat-sugar diet for 4 weeks in model group and curcumin group. The levels of blood glucose were determined 7 days after injection of STZ and rats with blood glucose levels more than 16.7 mM were used. Then each rat was supplemented with the appropriate dose of citrate buffer (normal and model group) or curcumin (Sigma-Aldrich) for 8 weeks. The normal group rats received neither STZ nor curcumin but only received the same volume of citrate buffer as the criteria of body mass. Curcumin group rats received curcumin at a dose of 300 mg/kg body weight per day by gavage. The dosages of animals were referred to the published literature (Soetikno et al. 2011). Water consumption and body weight were measured before and after administration. At 12 weeks, the rats were housed in metabolic cages (Jeungdo Bio & Plant Co., Seoul, Republic of Korea) for 24 h to collect urine. All rats were weighed and sacrificed under anaesthesia to obtain kidney and blood samples.

### Determination of biochemical indices

Spot urine and serum samples were collected immediately before sacrifice. Blood samples were collected from the retro-orbital plexus of rats under mild ether anaesthesia, in heparinized centrifuge tubes and immediately centrifuged at 2300 *g* for separation of plasma. Plasma was stored at 80 °C until assays were performed. The plasma was used for the estimation of glucose, blood urea nitrogen and creatinine with the appropriate kits purchased from Abcam (Cambridge, MA, USA). Commercial kits were used to measure urinary albumin (Bethyl Laboratories, Montgomery, AL, USA).

### Histopathological examination

Half of the kidneys were immediately snap-frozen in liquid nitrogen for subsequent protein extraction and enzymatic assays. The remaining excised kidneys were cut into about 2 mm thick transverse slices and fixed in 4% paraformaldehyde. After being embedded in paraffin, several transverse sections were obtained from the kidney and stained with haematoxylin–eosin (H&E) and Masson’s trichrome by standard procedures for glomerular sclerotic evaluation, and then were observed and photographed by light microscopy (Olympus, Tokyo, Japan).

### Transmission electron microscopy

Small pieces of renal cortex tissues were obtained and fixed in 2.5% glutaraldehyde. Then, they were washed with PBS and post-fixed with 1% osmium tetroxide. After gradient dehydration of acetone, the tissues were embedded in Araldite M (Sigma-Aldrich). Ultrathin sections were made using an ultramicrotome (Leica, Germany), and stained with uranyl acetate and lead citrate. The sections were examined through a transmission electron microscope (H-7700, Hitachi, Japan).

### Cell culture and treatment

Conditionally immortalized mouse podocytes (MPC5), purchased from Rantai Company (Shanghai, China), were cultured according to the previously described method (Guo et al. 2017). Briefly, podocytes were cultured in collagen I-coated dishes (BD Biosciences, Bedford, MA, USA) in RPMI 1640 medium (Invitrogen, Carlsbad, CA, USA) supplemented with 10% foetal bovine serum (FBS, Gibco BRL, Grand Island, NY, USA) 100 U/mL penicillin G, 100 µg/mL streptomycin (Gibco BRL), and 10 IU/mL of recombinant murine γ-interferon (IFN-γ, Invitrogen, Carlsbad, CA, USA) at 37 °C and 5% CO_2_. To induce differentiation, cells were treated for 14 days at 37 °C, and the medium was replaced with RPMI 1640 (containing 5% FBS without IFN-γ). The cells were then collected for the following assays.

Cells were divided into five groups: (1) control group: without any treatment; (2) model group: cells were incubated in medium with 10% serum from DN rats for 24 h to induce EMT; (3) curcumin group: cells were treated with serum from DN rats and curcumin; (4) curcumin + fumonisin B1 (Santa Cruz, Dallas, TX, USA) group: cells were treated with serum from DN rats, curcumin and fumonisin B1; (5) curcumin + 3-benzyl-5-[(2-nitrophenoxy) methyl]-dihydrofuran-2(3H)-one (3BDO) (Sigma-Aldrich) group: cells were treated with serum from DN rats, curcumin and 3BDO. And specifically podocytes were incubated in media with 40 µM curcumin for 24 h, or pre-treated with the Akt activator, 25 µM fumonisin B1 for 2 h (Wang et al. 2019), or co-treatment with the mTOR activator, 60 μM 3BDO (Sheng et al. 2017) in subsequent experiments to detect proteins expression.

### Western blot analysis

Renal tissues or podocytes cells were lysed in RIPA lysis buffer (P0013B, Beyotime Shanghai, China), supplemented with a complete protease inhibitor mixture (P1005, Beyotime Shanghai, China) and then centrifuged at 12,000 rpm for 30 min at 4 °C. The protein samples were mixed with loading buffer and then heated at 95 °C to 100 °C for 5 min. Separated proteins were transferred to a nitrocellulose membrane and blocked with 5% non-fat milk at room temperature for 1 h. The primary antibodies used for Western blotting were as follows: PI3K (CST, 1:1000), p-Akt (CST, 1:1000), p-mTOR (CST, 1:1000), LC3 (CST, 1:1000), p62 (CST, 1:2000), TWIST1 (CST, 1: 1000), E-cadherin (CST, 1:1000), vimentin (CST, 1:1000) and β-actin (CST, 1:3000). Blots were visualized by the enhanced chemiluminescence system (Santa Cruz Biotechnology Inc., Santa Cruz, CA, USA) and developed on the film.

### Statistical analysis

The experimental data were statistically analysed by one-way ANOVA using GraphPad Prism 6 (GraphPad Software, La Jolla, CA, USA). Statistically significant results by ANOVA were further analysed by Bonferroni *post hoc* analysis (where indicated). A *p*-value <0.05 was considered statistically significant. Error bars in the graphs were generated using mean ± SD values.

## Results

### Curcumin improves renal function and biochemical indexes in DN-induced rats

As shown in Table 1, the rats in the model group showed an obvious decrease in body weight and an increase in water intake compared with the normal group (*p* < 0.01). The weights in the curcumin-treated group were clearly raised and higher than that in the model (1.25 times) (*p* < 0.01), and water intakes were lower than that in the model (by 37.6%) (*p* < 0.01). Meanwhile, as shown in Table 2, blood glucose, serum creatinine, blood urea nitrogen and urine albumen in the model group were apparently increased and higher than that in the normal (*p* < 0.01). Curcumin reduced the levels of glucose, serum creatinine, blood urea nitrogen and urine albumen to some extent in DN-induced rats (by 28.4, 37.6, 33.5 and 22.4%, respectively), but still higher than that in the normal (*p* < 0.01).

**Table 1.** Comparisons of body weight and water intake in different groups after 8 weeks (x ± s, *n* = 10).

Group	Body weight (g)	Water intake (mL/24 h)
Normal	387.79 ± 3.85	16.75 ± 1.03
Model	242.05 ± 5.33**	172.34 ± 9.39**
Curcumin	304.63 ± 6.85**^##^	107.53 ± 9.16**^##^

Data were presented as means ± SD. **p < 0.01 vs. Normal group; ^##^*p* < 0.01 vs. Model group.

**Table 2.** Comparisons of blood glucose, serum creatinine, blood urea nitrogen and urine albumen in different groups after 8 weeks (x ± s, *n* = 10).

Group	Blood glucose (mM)	Serum creatinine (μM)	Urea nitrogen (mM)	Urine albumen (mg/24 h)
Normal	5.42 ± 0.16	25.24 ± 1.57	5.37 ± 0.22	5.82 ± 0.32
Model	29.11 ± 1.24**	80.68 ± 4.55**	27.65 ± 0.82**	23.20 ± 1.06**
Curcumin	19.35 ± 1.05**^##^	50.29 ± 4.34**^##^	18.37 ± 0.54**^##^	17.79 ± 1.03**^##^

Data were presented as means ± SD. **p < 0.01 vs. Normal group; ^##^*p* < 0.01 vs. Model group.

### Curcumin attenuates pathological changes in renal tissues of DN-induced rats

**Figure 1.**
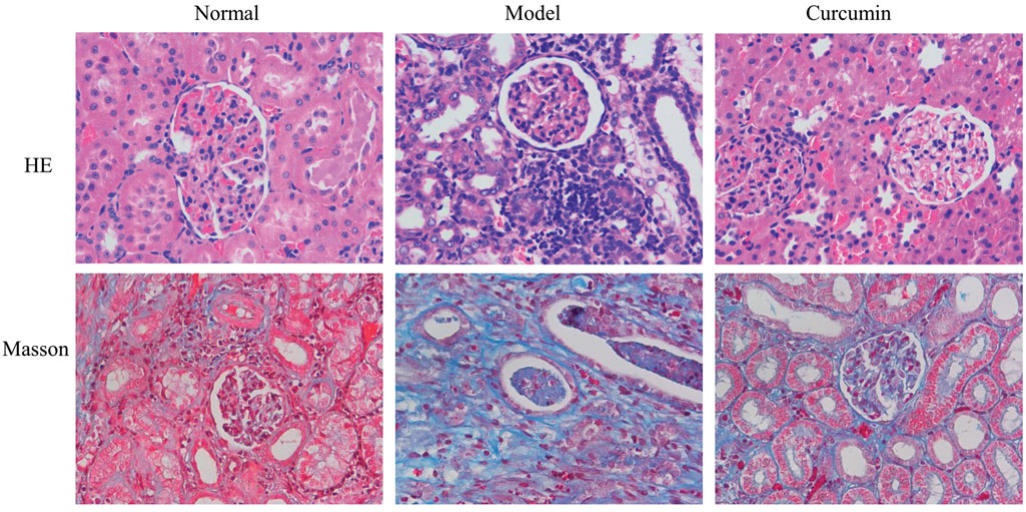
Effect of curcumin on histomorphological changes of renal tissues in DN-induced rats. Renal tissues were observed by staining with H&E and Masson and photographed by microscopy (bar: 400 µm).

As shown in Figure 1, HE staining revealed that the renal glomerular structure of rats in normal group was complete, the capillary cavity was uniform and consistent, the basement membrane was complete, the renal tubules were arranged neatly and the size was uniform, and no inflammatory cell infiltration was observed in the renal interstitium. Compared with the normal group, the model group showed obvious glomerular sclerosing, atrophy, obvious dilation of renal tubules, increased space, and a large number of inflammatory cell infiltration in renal interstitium. After treatment with curcumin, the pathological phenomena of kidney were significantly reduced, the glomeruli were not significantly atrophic, and the tubular dilatation and inflammatory cell infiltration were significantly reduced.

Masson staining of the kidney in rats was observed and the blue area represented the degree of renal fibrosis. Rats in the normal group only had a small amount of blue area in the renal interstitium, compared with the normal group, a large number of blue areas appeared in the uncle glomeruli, renal tubules and renal interstitium in model group, indicating severe renal fibrosis. After treatment with curcumin, the blue area of the kidney of the rats in the curcumin group decreased significantly, and there were some staining areas in the glomeruli and renal interstitium.

### Curcumin alters E-cadherin, vimentin and TWIST1 proteins in renal tissues of DN-induced rats

**Figure 2.**
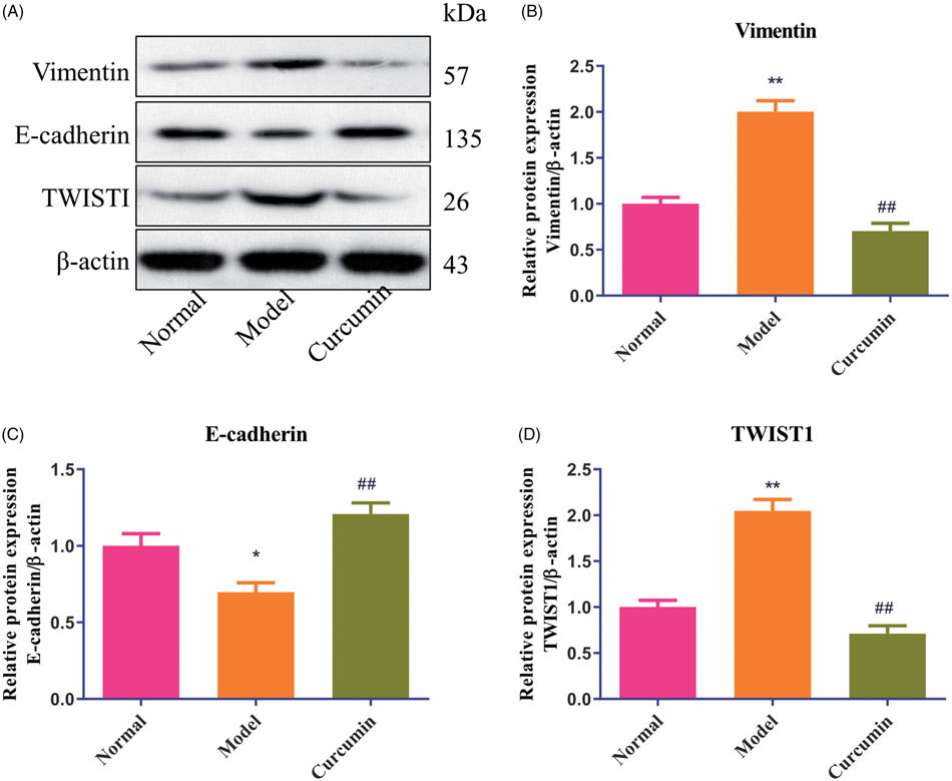
Effect of curcumin on E-cadherin, vimentin and TWIST1 proteins in renal tissues of DN-induced rats. (A) The levels of E-cadherin, vimentin and TWIST1 proteins from renal tissues were detected by Western blot and normalized to β-actin and then (B–D) relative band intensities were used in order to quantify E-cadherin, vimentin and TWIST1 protein. *p < 0.05, **p < 0.01 vs. the normal group, ^##^*p* < 0.01 vs. the model group.

As shown in Figure 2, compared with the normal group, the levels of vimentin and TWIST1 protein were increased about 2.0 times (*p* < 0.01), and E-cadherin protein was decreased in renal tissues of DN-induced rats (*p* < 0.05). Treatment with curcumin in DN-induced rats markedly upregulated the expression of E-cadherin protein (*p* < 0.01) (Figure 2(C)), downregulated the expression of vimentin and TWIST1 proteins (*p* < 0.01) (Figure 2(B,D)), and had no obvious difference with the normal group.

### Curcumin improves the autophagy in renal tissues of DN-induced rats

**Figure 3.**
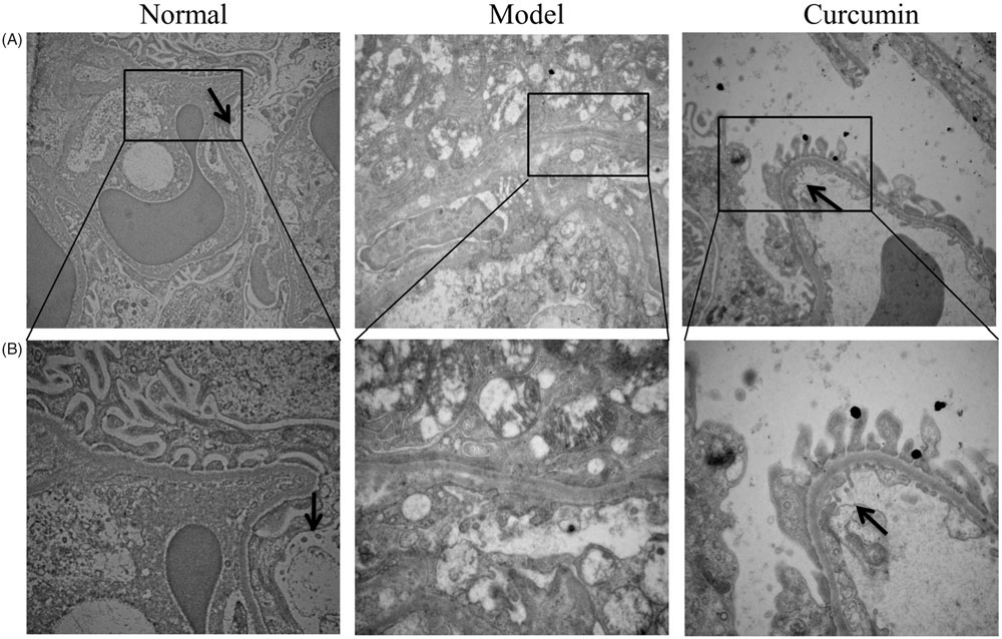
Effect of curcumin on autophagic vacuoles in renal tissues of DN-induced rats. Autophagic vacuoles in renal tissues were observed (black arrow) and photographed by transmission electron microscopy (bar: A – 15,000 µm; B – 30,000 µm).

In the renal electron microscope images of the normal group (Figure 3), the glomerular podocytes were evenly distributed without clear thickening structure. In the model group, the glomerular podocyte showed obvious thickening and structure became fuzzy, and the podocyte foot process was out of shape and showed different degrees of fusion, autophagic vacuoles were not present. Compared with the normal group and the model group, podocytes in curcumin group increased but to a lesser extent than the model group, and podocyte foot process was partially fused; meanwhile, autophagic vacuoles were observed.

### Curcumin alters the autophagy-related proteins and PI3K/Akt in renal tissues of DN-induced rats

**Figure 4.**
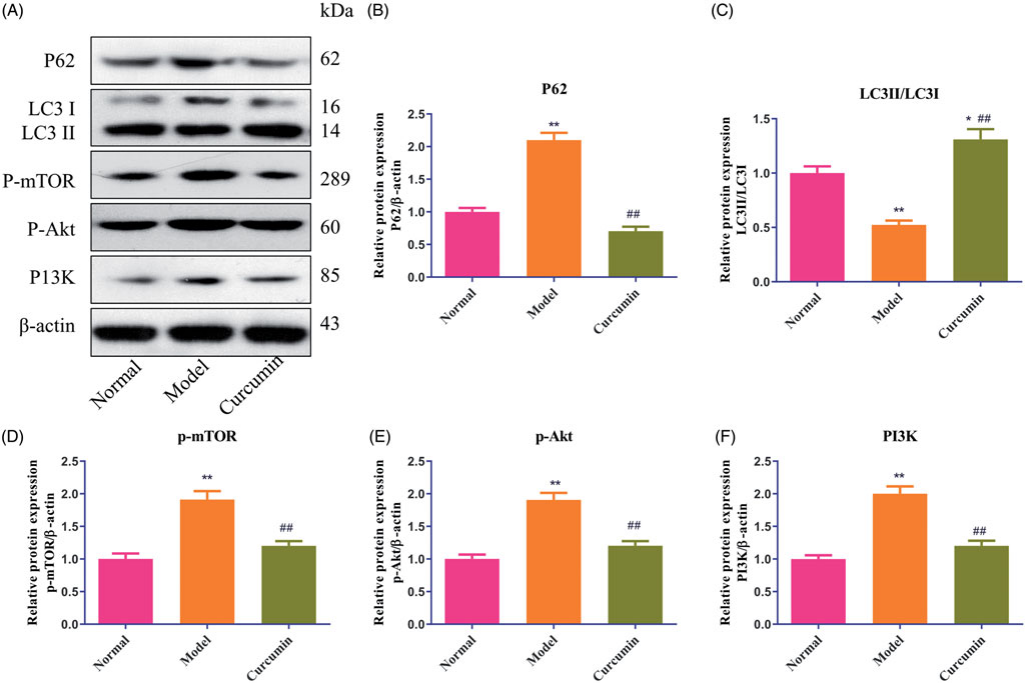
Effect of curcumin on p62, LC3, P-mTOR, p-Akt and P13K proteins in renal tissues of DN-induced rats. (A) The levels of p62, LC3, P-mTOR, p-Akt and P13K proteins from renal tissues were detected by Western blot and normalized to β-actin and then (B–F) relative band intensities were used in order to quantify p62, LC3, p-mTOR, p-Akt and P13K proteins. *p < 0.05, **p < 0.01 vs. the normal group, ^##^*p* < 0.01 vs. the model group.

As shown in Figure 4, compared with the normal group, the levels of p62 and p-mTOR protein were increased (*p* < 0.01) and LC3 protein was decreased in the model group (*p* < 0.05). Treatment with curcumin markedly upregulated the expression of LC3 protein and had obvious differences with the normal group (*p* < 0.05) and model group (*p* < 0.01) (Figure 4(C)). The expression of p62 and p-mTOR proteins in curcumin-treated rats were lower than the model group (*p* < 0.01) and had no difference with the normal group (Figure 4(B,D)). Meanwhile, compared with the normal group, the levels of p-Akt and P13K protein were increased (*p* < 0.01) in DN-induced rats, and curcumin effectively inhibited the expression of p-Akt and P13K proteins (*p* < 0.01) (Figure 4(E,F)).

### Curcumin alters autophagy in MPC5 cells

**Figure 5.**
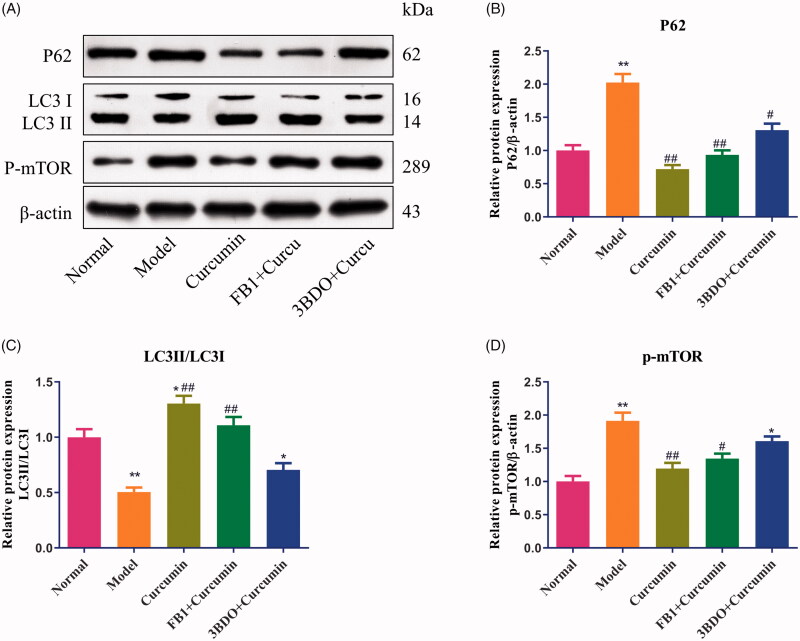
Effect of curcumin on p62, LC3 and p-mTOR proteins in MPC5 cells. (A) EMT-induced MPC5 cells were established and cultured in curcumin with or with fumonisin B1 or 3BDO. The levels of p62, LC3 and p-mTOR proteins from renal tissues were detected by Western blot and normalized to β-actin and then (B–D) relative band intensities were used in order to quantify p62, LC3 and p-mTOR proteins. *p < 0.05, **p < 0.01 vs. the normal group, ^#^*p* < 0.05, ^##^*p* < 0.01 vs. the model group.

MPC5 cells can be used as a cell model to investigate podocyte apoptosis or autophagy (Liu et al. 2017). As shown in Figure 5, compared with the normal group, the levels of p62 and p-mTOR protein were increased (*p* < 0.01), and LC3 protein was decreased in EMT-induced MPC5 cells (*p* < 0.01). Treatment with curcumin at 40 µM after inducing EMT in cells markedly upregulated the expression of LC3 protein (Figure 5(C)) and downregulated the expression of p62 and p-mTOR proteins (*p* < 0.01) (Figure 5(B,D)) (*p* < 0.01), which were consistent with the results of *in vivo* experiments.

Then EMT-induced MPC5 cells were exposed to curcumin combined with an Akt inducer, fumonisin B1 or the mTOR stimulator, 3BDO. Fumonisin B1 or 3BDO elevated the levels of p62 and p-mTOR protein expression and lowered the LC3 protein expression, and reversed the effect of curcumin to some extent.

### Curcumin alters E-cadherin, vimentin and TWIST1 proteins in MPC5 cells

**Figure 6.**
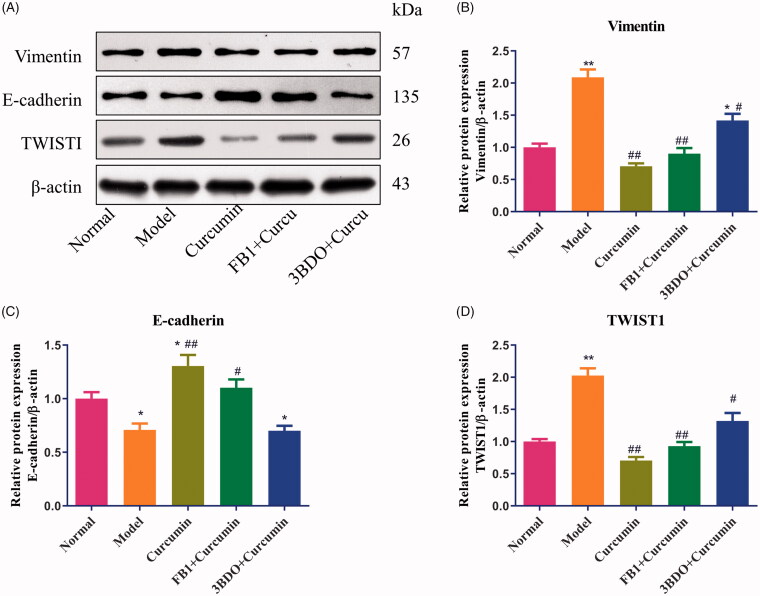
Effect of curcumin on E-cadherin, vimentin and TWIST1 proteins in MPC5 cells. (A) EMT-induced MPC5 cells were established and cultured in curcumin with or with fumonisin B1 or 3BDO. The levels of E-cadherin, vimentin and TWIST1 protein from MPC5 cells were detected by Western blot and normalized to β-actin and then (B–D) relative band intensities were used in order to quantify E-cadherin, vimentin and TWIST1 protein. *p < 0.05, **p < 0.01 vs. the normal group, ^##^*p* < 0.05, ^##^*p* < 0.01 vs. the model group.

As shown in Figure 6, as we expected, compared with the normal group, the levels of vimentin and TWIST1 protein were increased (*p* < 0.01), and E-cadherin protein was decreased in EMT-induced MPC5 cells (*p* < 0.05). Treatment with curcumin in EMT-induced MPC5 cells markedly upregulated the expression of E-cadherin protein (Figure 6(C)) and downregulated the expression of vimentin and TWIST1 proteins (*p* < 0.01) (Figure 6(B,D)) (*p* < 0.01), Curcumin combined with fumonisin B1 or 3BDO elevated the levels of vimentin and TWIST1 proteins and lowered the E-cadherin protein, indicating that the inhibiting effect of curcumin on EMT would be hampered.

### Curcumin alters the autophagy-related proteins and PI3K/Akt in MPC5 cells

**Figure 7.**
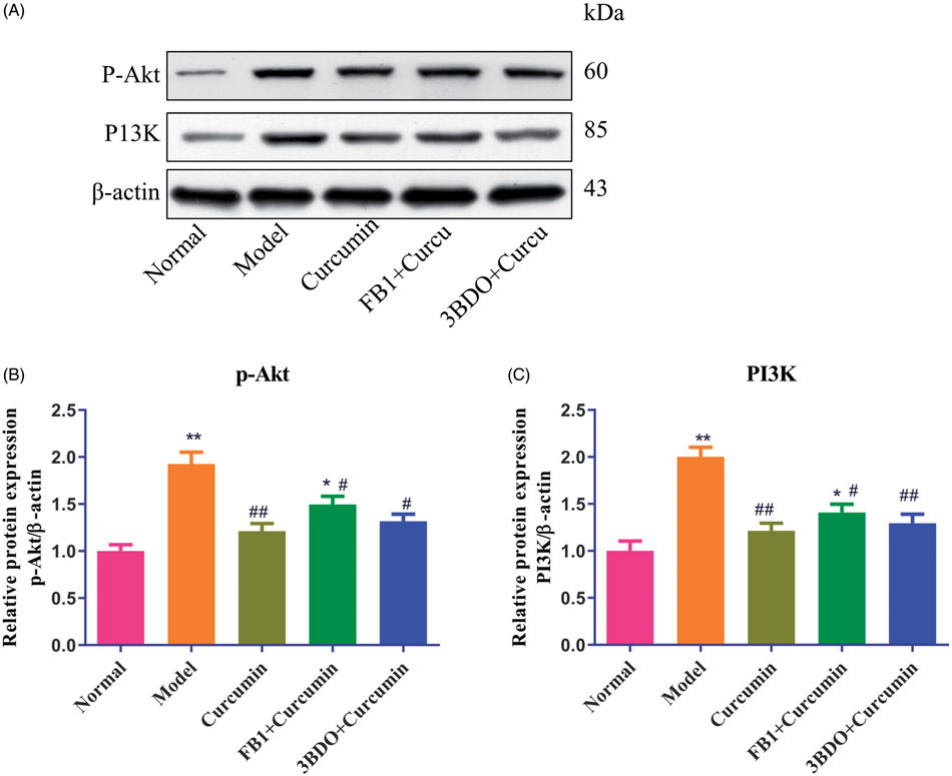
Effect of curcumin on PI3K and p-Akt proteins in MPC5 cells. (A) EMT-induced MPC5 cells were established and cultured in curcumin with or with fumonisin B1 or 3BDO. The levels of PI3K and p-Akt proteins from renal tissues were detected by Western blot and normalized to β-actin and then (B–C) relative band intensities were used in order to quantify PI3K and p-Akt proteins. *p < 0.05, **p < 0.01 vs. the normal group, ^#^*p* < 0.05, ^##^*p* < 0.01 vs. the model group.

As shown in Figure 7, compared with the normal group, the levels of p-Akt and P13K proteins were increased (*p* < 0.01) in EMT-induced MPC5 cells. Treatment with curcumin in EMT-induced MPC5 cells effectively downregulated the expression of p-Akt and P13K proteins (*p* < 0.01) (Figure 7(B,C)) (*p* < 0.01), Curcumin combined with fumonisin B1 or 3BDO elevated the levels of p-Akt and P13K proteins, which to some extent reverse the effect of curcumin.

## Discussion

DN is characterized by a plethora of signalling abnormalities. Recent trials have suggested that intensive glucose-lowering treatment leads to hypoglycaemic events, which can be dangerous. Curcumin is the active ingredient of turmeric, which has been widely used in many countries for centuries to treat numerous diseases owing to its antioxidant and anti-inflammatory activities. This pleiotropic regulation of redox balance of cell and inflammation might be the basis of curcumin’s beneficial activities in various pathologic conditions including diabetic complications (Jeenger et al. 2015). Chronic treatment with curcumin significantly attenuated both renal dysfunction and oxidative stress in diabetic rats (Sharma et al. 2006; Soetikno et al. 2013). In our experiment, firstly the rat model of DN induced by streptozotocin and high-fat and high-sugar diet was established. Treatment with curcumin at 300 mg/kg/d effectively improved renal function, and reduced the levels of glucose, serum creatinine, urea nitrogen and urine albumen, histomorphological changes were attenuated in DN rats, which were identical with those reported in the literature (Wu et al. 2014). On the other hand, podocyte foot process was partially fused in renal tissues of DN rats after treatment with curcumin.

Podocyte is the inherent cells of kidney. As the most vulnerable cell component of glomeruli, podocytes play an important role in the structure and function of glomeruli through the cytoskeleton system dominated by actin and the protein molecules specifically expressed by podocytes. Podocyte injury is a major cause of nephrotic proteinuria and glomerular sclerosis. PI3K-Akt/mTOR is an important signalling pathway existing in podocytes, which has a certain role in the regulation of autophagy of podocyte. It was reported that curcumin was beneficial in ameliorating the development of DN through inhibition of PKC-α and PKC-β1 activity-ERK1/2 pathway (Soetikno et al. 2011) or suppression NLRP3 inflammasome signalling (Lu et al. 2017). However, PI3K-Akt/mTOR pathway has not been discussed in treatment the DN with curcumin. Curcumin exerts a renoprotective effect in the presence of glycation or glycoxidation end products, at least in part by activating protective autophagy in tubular epithelial NRK-52E cells (Wei et al. 2017). And the classic autophagy pathways such as Atg5, Atg7, mTOR and LC3 were revealed to be involved in the autophagy dysfunction in podocytes (Liu et al. 2017). Here, we observed that autophagic vacuoles were increased in renal tissues of DN rats after treatment with curcumin. Then treatment with curcumin showed an increase in the level of LC3 protein, and a decreased in the levels of p62, p-mTOR, p-Akt and P13K proteins in DN rats and MPC5 cells. In addition, fumonisin B1, an Akt activator or 3BDO, a mTOR inducer led a decrease in level of LC3 protein and an increase in p62, p-mTOR, p-Akt and P13K proteins in curcumin-treated MPC5 cells, which hinted that curcumin regulates autophagy in mouse podocytes via PI3K/Akt/mTOR pathway.

Emerging evidence suggests that diabetes also alters the phenotype of normal, non-fibroblast kidney cells, such as mesangial cells, tubular epithelial cells, and bone marrow-derived progenitors. Experiments have shown that cytokines, high glucose, and advanced glycation end products induced profibrotic changes in kidney cell phenotype by the processes of myofibroblast transdifferentiation and EMT (Peng et al. 2014). And curcumin at doses of 1, 5 and 10 μM dose-dependently attenuated high glucose-induced podocyte apoptosis by regulating functional connections between caveolin-1 phosphorylation and ROS in MPC5 cells (Sun et al. 2016). Curcumin also ameliorated podocytic adhesive capacity damage under mechanical stress by inhibiting miR-124 expression (Li et al. 2013). Podocytes are visceral epithelial cells. When podocytes undergo EMT, they lose epithelial-like phenotypic markers of mature podocytes, such as E-cadherin, and upregulated mesenchymal cell-like phenotypic markers such as vimentin and TWIST1. Curcumin prevented EMT of podocytes, proteinuria, and kidney injury in DN by suppressing the phosphorylation of caveolin-1, and increasing stabilization of caveolin-1 and β-catenin (Sun et al. 2014). As we expected, curcumin upregulated the levels of E-cadherin protein, and downregulated the levels of vimentin and TWIST1 proteins in DN rats as well as in MPC5 cells. In addition, fumonisin B1 and 3BDO led a decrease in levels of E-cadherin protein and an increase in vimentin and TWIST1 proteins in curcumin-treated MPC5 cells, suggesting that curcumin could suppress mesenchymal trans-differentiation of podocytes through PI3K/Akt/mTOR pathway.

## Conclusions

The present study showed that curcumin could regulate autophagy and inhibit mesenchymal trans-differentiation of DN foot cells via PI3K/Akt/mTOR pathway *in vivo* and *in vitro*. These findings strengthen the therapeutic rationale for curcumin use in DN treatment and could also be beneficial in the therapy for other complications of DN.
